# Distributions and relationships of virio- and picoplankton in the epi-, meso- and bathypelagic zones of the Amundsen Sea, West Antarctica during the austral summer

**DOI:** 10.3389/fmicb.2022.941323

**Published:** 2022-07-27

**Authors:** Meiaoxue Han, Guangfu Luo, Jianfeng He, Yantao Liang, Xuechao Chen, Gang Liu, Yue Su, Fuyue Ge, Hao Yu, Jun Zhao, Qiang Hao, Hongbing Shao, Yeong Yik Sung, Wen Jye Mok, Li Lian Wong, Andrew McMinn, Min Wang

**Affiliations:** ^1^College of Marine Life Sciences, Key Lab of Polar Oceanography and Global Ocean Change, Institute of Evolution and Marine Biodiversity, and Frontiers Science Center for Deep Ocean Multispheres and Earth System, Ocean University of China, Qingdao, China; ^2^Antarctic Great Wall Ecology National Observation and Research Station, Polar Research Institute of China, Shanghai, China; ^3^MNR Key Laboratory for Polar Science, Polar Research Institute of China, Shanghai, China; ^4^College of Environmental Science and Engineering, Tongji University, Shanghai, China; ^5^UMT-OUC Joint Centre for Marine Studies, Qingdao, China; ^6^Key Laboratory of Marine Ecosystem Dynamics, Second Institute of Oceanography, Ministry of Natural Resources, Hangzhou, China; ^7^Institute of Marine Biotechnology, University of Malaysia Terengganu (UMT), Kuala Terengganu, Malaysia; ^8^Institute for Marine and Antarctic Studies, University of Tasmania, Hobart, TAS, Australia; ^9^Department of Critical Care Medicine, The Affiliated Hospital of Qingdao University, Qingdao, China

**Keywords:** polynya, deep sea, picoplankton, virioplankton, Amundsen Sea, upwelling

## Abstract

Virioplankton and picoplankton are the most abundant marine biological entities on earth and mediate biogeochemical cycles in the Southern Ocean. However, understanding of their distribution and relationships with environmental factors is lacking. Here, we report on their distribution and relationships with environmental factors at 48 stations from 112.5° to 150°W and 67° to 75.5°S in the Amundsen Sea of West Antarctica. The epipelagic stations were grouped into four clusters based on the virio- and picoplankton composition and abundance. Clusters three and four, which were associated with the ice-edge blooms in the coastal and Amundsen Sea Polynya (ASP) areas, had high abundances of autotrophic picoeukaryotes; this resulted in subsequent high abundances of heterotrophic prokaryotes and viruses. Cluster two stations were in open oceanic areas, where the abundances of autotrophic and heterotrophic picoplankton were low. Cluster one stations were located between the areas of blooms and the oceanic areas, which had a low abundance of heterotrophic prokaryotes and picoeukaryotes and a high abundance of virioplankton. The abundance of viruses was significantly correlated with the abundances of autotrophic picoeukaryotes and Chl-*a* concentration in oceanic areas, although this reflected a time-lag with autotrophic picoeukaryote and heterotrophic prokaryotes abundances in ice-edge bloom areas. The upwelling of Circumpolar Deep Water (CDW) might have induced the high abundance of autotrophic picoeukaryotes in the epipelagic zone, and the sinking particulate organic carbon (POC) might have induced the high abundance of heterotrophic prokaryotes and virioplankton in the meso- and bathypelagic zones. This study shows that the summer distribution of virio- and picoplankton in the Amundsen Sea of West Antarctica was mainly controlled by upwelling of the CDW and the timing of ice-edge blooms.

## Introduction

Virio- and picoplankton (0.2–2.0 μm), which includes autotrophic picoeukaryotes and heterotrophic prokaryotes, are arguably the most abundant and diverse biological entities in the ocean (Bergh et al., 1989; Arrigo and van Dijken, 2003; Azam and Malfatti, 2007) and have major roles in marine biogeochemical cycles (Weinbauer, 2004; DeLong and Karl, 2005; Brussaard et al., 2008; Aylward et al., 2017). Marine viruses can regulate the microbial community to maintain microbial diversity (Weitz et al., 2005; Rohwer and Thurber, 2009; Payet and Suttle, 2013; Paez-Espino et al., 2016) through different ecological strategies, such as ‘kill the winner,’ whereby the host cells are lysed, and ‘piggyback the winner’ where viruses coexist with their hosts (Thingstad, 2000; Weitz and Dushoff, 2007; Rodriguez-Brito et al., 2010; Thingstad et al., 2014; Knowles et al., 2016). Viruses can also influence the cycling and sequestration of organic matter through the ‘viral shunt’ and ‘viral shuttle’ (Wilhelm and Suttle, 1999; Zimmerman et al., 2020). Recently, a study reported that viral lysis was a major loss factor, responsible for roughly half (58%) of seasonal phytoplankton carbon losses in the Southern Ocean (Biggs et al., 2021). Heterotrophic prokaryotes (including bacteria and archaea) are key components of the microbial food web and play critical roles in the utilization, transformation, remineralization and sequestration of dissolved and particulate organic matter (Azam and Malfatti, 2007; Jiao et al., 2010). Heterotrophic prokaryotes are abundant and a major pathway for carbon flow although the low water temperature and extremely variable seasonal productivity in the Southern Ocean (Leakey et al., 1996; Rivkin et al., 1996; Delille, 2004). Autotrophic picophytoplankton are dominant and ubiquitous primary producers in many areas of the epipelagic zone (0–200 m below the surface) and can account for >50% of the marine primary production (Brown et al., 1999; Worden et al., 2004, 2015). In the Southern Ocean, autotrophic picophytoplankton contribute 20–40% and even up to 70% of the chlorophyll *a* biomass occasionally (Agawin et al., 2002; Hewes, 2009; Lin et al., 2012).

Much of Antarctica, especially the Amundsen Sea, is characterized by a narrow continental shelf, persistent sea ice, and coastal polynyas located close to large ice shelves (Nihashi and Ohshima, 2015). Satellite images show that the Amundsen Sea has experienced significant surface warming and loss of sea ice over the last few decades (Jacobs et al., 1996; Jenkins et al., 1997; Stammerjohn et al., 2008; Thoma et al., 2008). The glaciers near the Amundsen Sea are undergoing the highest rates of glacial retreat and thinning on the Antarctic continent (Rignot, 2008; Schoof, 2010). At the start of spring, sea ice, which contains high iron concentrations, retreats (Poulton and Raiswell, 2005) resulting in an increase in irradiance, allowing the formation of phytoplankton blooms near the coast, especially in the coastal polynyas (Alderkamp et al., 2012; Fragoso and Smith, 2012; Gerringa et al., 2012). The Amundsen Sea Polynya (ASP) is reported to be the most productive of polynyas around Antarctica (Arrigo and van Dijken, 2003; Arrigo et al., 2012), and is regarded as an important sink for atmospheric CO_2_ (Arrigo et al., 2008). Thus, determining the role of microbes that are associated with this primary production (PP) in the productive polynyas and continental shelves in the Southern Ocean is particularly urgent (Ducklow and Yager, 2007; Kirchman et al., 2009; Hyun et al., 2016; Swalethorp et al., 2019).

To date, the spatial and temporal variability of viral abundance and structure in the Southern Ocean is mostly limited to the western Antarctic Peninsula area (Vaque et al., 2017; Sotomayor-Garcia et al., 2020; Evans et al., 2021), eastern Prydz Bay (Liang et al., 2016), the Cosmonaut and Cooperation Seas (Han et al., 2022) and coastal areas (Marchant et al., 2000; Pearce et al., 2007). Understanding of the abundance and distribution of these groups in the Amundsen Sea of West Antarctic is poor. Only one study has reported on microbial heterotrophy in the ASP, and this documented the viral response to a *Phaeocystis antarctica* bloom; however, only the upper 100 m of the water column was investigated (Williams et al., 2016). Importantly, understanding how the environment regulates virus dynamics and virus–host interactions on a large-scale is still poor, especially in the deep sea.

Here we report on the large-scale horizontal and vertical distribution of virio- and picoplankton in the Amundsen Sea of West Antarctica in summer and explore their relationships with environmental factors. The study included 48 stations where the entire water column was sampled (from the surface to 4,725 mbs). The main environmental factors influencing viral dynamics were identified and a framework of the viral roles in ice-edge blooms was given.

## Materials and methods

### Study area and sampling

**Figure 1:**
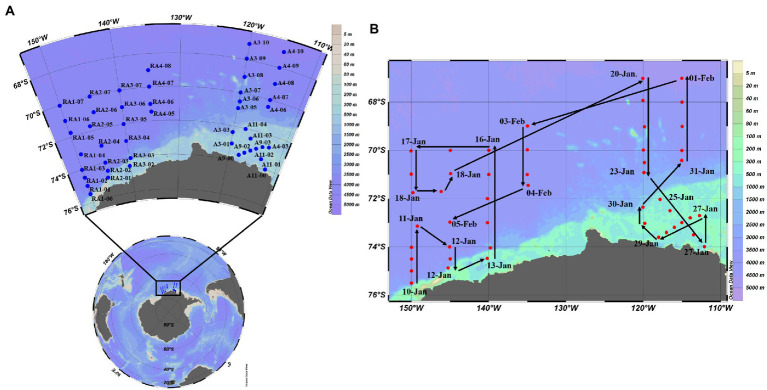
Sampling stations **(A)** and voyage track **(B)** in the Amundsen Sea of West Antarctica.

Samples were collected from 8 transects at up to 14 depths within the epi-, meso- and bathypelagic zones in the Amundsen Sea of West Antarctica (35–78°E, 62–70°S) from 10th January to 5th February in 2020 aboard R/V XUELONG (Figure 1). Seawater was sampled with 10-L Niskin bottles attached to a rosette equipped with a Seabird 911Plus CTD (Bellevue, United States), including temperature, salinity, density and dissolved oxygen (DO). Triplicate samples (1.5 ml) for virio- and picoplankton abundance were fixed with glutaraldehyde (final concentration: 0.5%), frozen in liquid nitrogen and stored at −80°C until analysis (Brussaard, 2004). Seawater was collected with Niskin bottles for nutrient concentrations (SiO_3_, PO_4_, NH_4_, NO_3_ and NO_2_), stored in acid-washed and MilliQ water rinsed Nalgene bottles at −20°C and analyzed using a four-channel continuous flow Technicon AA3 Auto-Analyzer (Whitledge et al., 1981; Bran and Luebbe, 1997). Chlorophyll *a* (Chl-*a*) concentration *in situ* was determined by fluorescence (Mock and Hoch, 2005). Seawater was collected with Niskin bottles and were filtered onto Whatman GF/F filters (0.7 mm nominal pore size). The pigments were extracted in 90% acetone overnight at 4°C. The concentration of pigments was measured on a Turner Designs 10-AU field fluorometer, calibrated with a purified Chl-*a* standard (Sigma-Aldrich, Germany). Eight-days-averaged sea ice concentrations were obtained from the AMSR2 dataset at the University of Bremen, with a spatial resolution of 6.25 km × 6.25 km.^1^ Eight-days-averaged concentration of Chl-*a* with a spatial resolution of 4 km × 4 km was retrieved from the products generated by the Ocean Color component of the European Space Agency Climate Change Initiative project.^2^

### Virio- and picoplankton counting using flow cytometry

Viral abundance was estimated following previously published methods (Brussaard, 2004; Liang et al., 2016) with some modifications. The fixed and frozen samples (−80°C) were thawed at 37°C, diluted 100-fold with 0.02 μm filtered Tris-EDTA buffer (pH = 8, Sigma-Aldrich, Germany) and stained with SYBR Green I (final concentration of 0.5 × 10^−4^ of the Molecular Probes stock solution, Thermo Fisher, United States) in the dark at 80°C. The incubated samples were then cooled at room temperature for 5 min and analyzed with a CytoFLEX flow cytometer (Beckman, China) at a total volume of 30 μl sample^−1^.

For bacterial enumeration (Marie et al., 1997; Liang et al., 2016), the thawed samples were diluted 10 fold with 0.02 μm filtered Tris-EDTA buffer, stained with SYBR Gold (final dilution of 10^−4^ of the commercial stock solution) for 15 min in the dark, and analyzed with the CytoFLEX flow cytometer (Beckman, China) for 1 min at a delivery rate of 60 μl min^−1^.

Picoeukaryotic abundance was determined following the method of Jiao et al. (2002), Wang et al. (2010), and Liang et al. (2014), and identified from plots of side scatter versus red fluorescence and orange fluorescence. 2 μm diameter fluorescent microspheres (Polysciences Inc., Warrington, United States) were added to all samples as an internal standard to calibrate flow rate and cell size, then run for 2 min at a rate of 60 μl min^−1^ with a red fluorescence filter set. The measurements were performed on a Beckman Coulter CytoFLEX™ flow cytometer at a wavelength of 488 nm and with the standard filter setup.

The ratios of viruses and their potential host cell abundances were calculated, including the ratio of viral and picoeukaryotic abundance (VEukR), the ratio of viral and heterotrophic prokaryotic abundance (VPR; Liang et al., 2017).

### Statistical analysis

One-way ANOVAs were used to evaluate variations between different samples using SPSS 18.0 software (SPSS Inc., Chicago, United States). Cluster analysis was conducted based on the Euclidean distance to determine dissimilarity in composition and abundance. Pearson correlation analysis among virio-, picoplankton and environmental factors was conducted using R packages psych and vegan.^3^ Redundancy analysis (RDA) was conducted to investigate the relationships between virio-, picoplankton and environmental variables in using CANOCO 4.5 software (Microcomputer Power, Ithaca, United States). The Euclidian distance-based multivariate analysis for a linear model using forward selection (DISTLM forward) was performed to access the relationships among virio-, picoplankton and environmental factors (Gastrich et al., 2004). In the epipelagic (0–200 m) zone, 14 variables were used to explain the variations in viral abundance, including longitude, latitude, depth, salinity, temperature, DO, SiO_3_, PO_4_, NO_3_, NO_2_, NH_4_, Chl-*a*, abundance of picoeukaryotes and heterotrophic prokaryotes. As picoeukaryotes and Chl-*a*, were undetectable in the meso- (200–1,000 m) and bathypelagic (1,000–4,725 m) zones, only 12 variables were used to explain the variations in viral abundances, including longitude, latitude, depth, salinity, temperature, DO, SiO_3_, PO_4_, NO_3_, NO_2_, NH_4_ and abundance of heterotrophic prokaryotes. Where necessary, the data were logarithmically transformed to reduce the influence of extreme values on normal data distribution.

## Results

### Hydrological conditions

Increased Chl-*a* concentrations were closely correlated with sea ice retreat (Supplementary Figures S1, S2). In general, the sea ice in the ASP melted first (about in mid-November), corresponding with an increase in concentration of Chl-*a* at this time; it then remained at a relatively high concentration until the beginning of February. From the beginning of December, the sea ice in the western coastal areas to 73°S began to melt and the concentration of Chl-*a* increased accordingly. The other survey areas have a relative low concentration of Chl-*a* (Supplementary Figure S2). The average water temperature was −0.93 ± 1.00, 0.89 ± 1.16 and 0.51 ± 0.20°C in the epi-, meso- and bathypelagic zones, respectively (Table 1). The average salinity was lower in the epipelagic zone, due to sea ice melt, than that in meso- and bathypelagic zones (ANOVA, *p* < 0.0001, Table 1).

**Table 1:** Environmental factors, viral abundance and ratios between virus and specific picoplankton potential host lineages in the water columns of the Amundsen Sea of West Antarctica.

	Parameters	Epipelagic (*n* = 360)	Mesopelagic (*n* = 136)	Bathypelagic (*n* = 105)
Temperature (°C) (*p* < 0.0001)	Average	−0.93	0.89	0.51
	s.d.	1.00	1.16	0.20
	Median	−1.41	1.38	0.45
	Min	−1.81	−1.78	0.24
	Max	2.04	2.02	1.04
Salinity (‰) (*p* < 0.0001)	Average	33.99	34.59	34.70
	s.d.	0.33	0.20	0.01
	Median	34.06	34.70	34.70
	Min	32.23	34.12	34.69
	Max	34.65	34.73	34.72
DO (mg L^−1^) (*p* < 0.0001)	Average	10.25	6.69	6.83
	s.d.	1.72	1.00	0.15
	Median	9.95	6.27	6.83
	Min	5.96	5.73	6.35
	Max	15.59	9.66	7.09
Picoeukaryotes (×10^3^ cells ml^−1^)	Average	2.50	ND	ND
	s.d.	2.31	ND	ND
	Median	2.11	ND	ND
	Min	0.01	ND	ND
	Max	12.95	ND	ND
Heterotrophic prokaryotes (×10^5^ cells ml^−1^) (*p* < 0.0001)	Average	1.82	0.80	0.81
	s.d.	2.02	0.54	0.88
	Median	1.17	0.60	0.50
	Min	0.14	0.16	0.03
	Max	18.22	2.93	5.97
Viral abundance (×10^7^ VLPs ml^−1^) (*p* < 0.0001)	Average	1.42	1.05	0.78
	s.d.	1.28	1.12	0.66
	Median	1.03	0.77	0.60
	Min	0.00	0.05	0.00
	Max	10.16	9.85	3.17
VPR (*p* = 0.01)	Average	126.40	171.72	154.26
	s.d.	133.62	170.08	191.59
	Median	96.60	110.10	101.56
	Min	0.01	2.03	13.95
	Max	1,066.70	920.77	1554.85
VEukR (×10^3^)	Average	70.03	ND	ND
	s.d.	565.75	ND	ND
	Median	5.80	ND	ND
	Min	0.00	ND	ND
	Max	10,160.80	ND	ND

ND, not detected.

**Figure 2:**
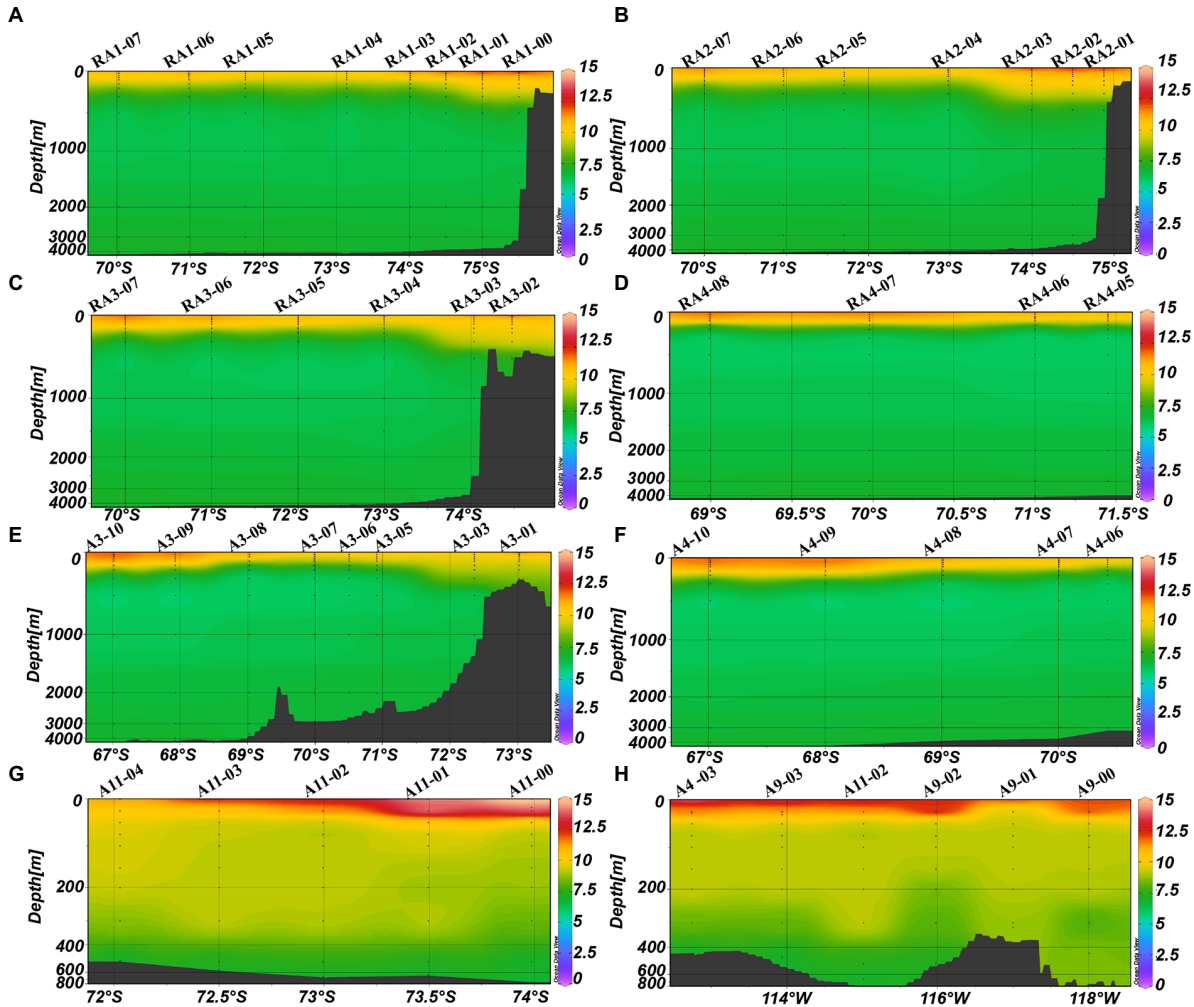
Distributions of DO (mg L^−1^) in the Amundsen Sea of West Antarctica. **(A)** RA1 transect, **(B)** RA2 transect, **(C)** RA3 transect, **(D)** RA4 transect, **(E)** A3 transect, **(F)** A4 transect, **(G)** A11 transect, and **(H)** A9 transect.

The average DO was higher in the epipelagic zone than that in the meso- and bathypelagic zones (ANOVA, *p* < 0.0001, Table 1; Figure 2). And in RA transects (including transects RA1, RA2 and RA3), the DO was higher in the coast than that in the oceanic areas (Figure 2), whereas the transects A3 and A4 had the opposite trend (Figure 2). DO was not significantly different between the four clusters (*p* = 0.918) in the epipelagic zone.

### Distribution of virioplankton

**Figure 3:**
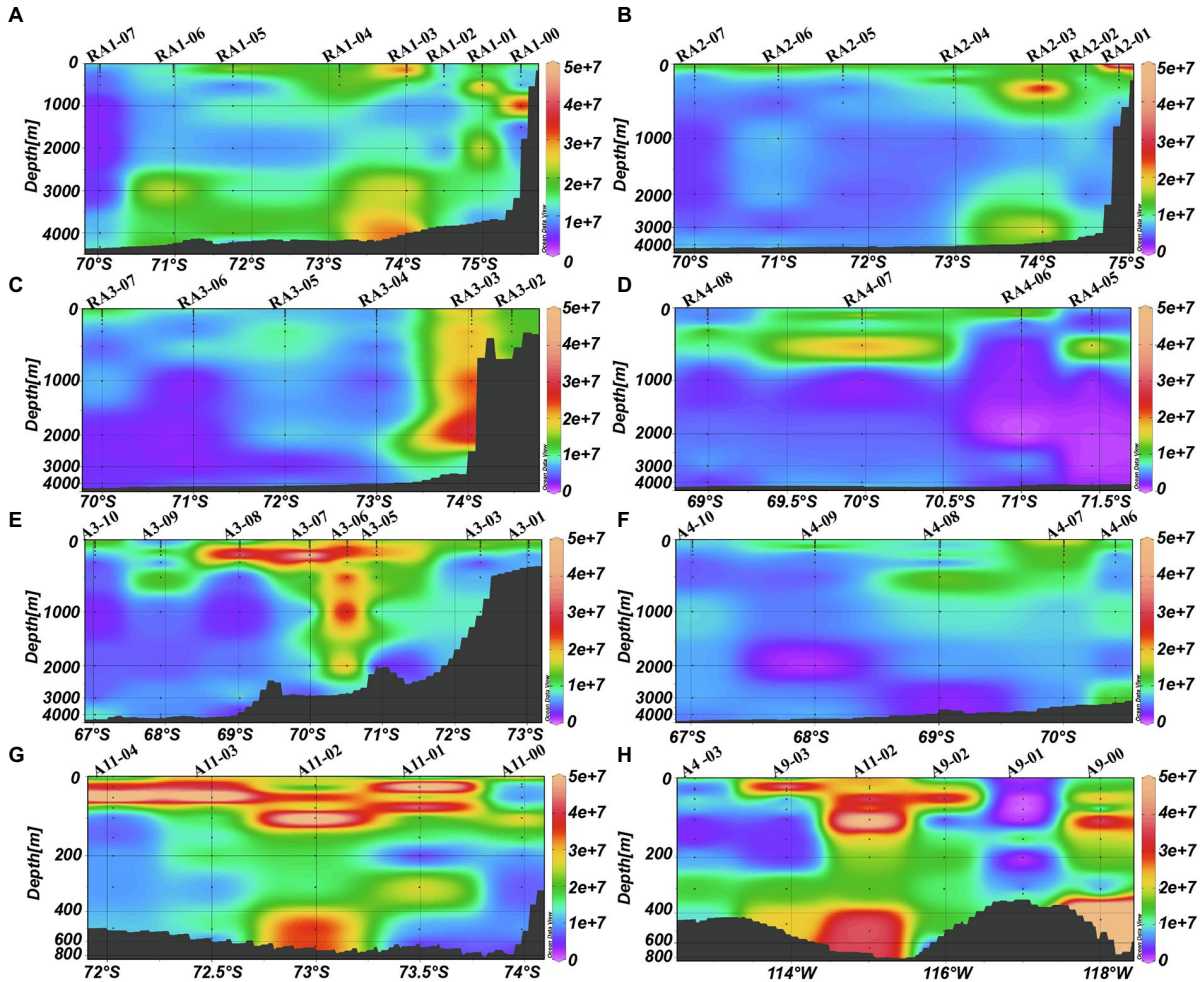
Distributions of viral abundance (VLP ml^−1^) in the Amundsen Sea of West Antarctica. **(A)** RA1 transect, **(B)** RA2 transect, **(C)** RA3 transect, **(D)** RA4 transect, **(E)** A3 transect, **(F)** A4 transect, **(G)** A11 transect, and **(H)** A9 transect.

Viral abundances decreased from the surface to the bottom (ANOVA, *p* < 0.001, Table 1; Figure 3). The depth-averaged viral abundances were 1.42 ± 1.28 × 10^7^, 1.05 ± 1.12 × 10^7^ and 0.78 ± 0.66 × 10^7^ virus-like particles (VLPs) ml^−1^ in the epi-, meso- and bathypelagic zones, respectively (Table 1). In RA1, RA2, RA3, A3, A9 and A11 transects, some high viral abundances were also detected in the meso- and bathypelagic zones (Figures 3A–C,E,G,H).

The average virus-to-heterotrophic prokaryotes ratios (VPR) were higher in the meso- and bathypelagic zones (171.72 ± 170.08 and 154.26 ± 191.59, respectively) than that in the epipelagic zone (126.40 ± 133.62, ANOVA, *p* = 0.01, Table 1).

### Distribution of picoplankton

**Figure 4:**
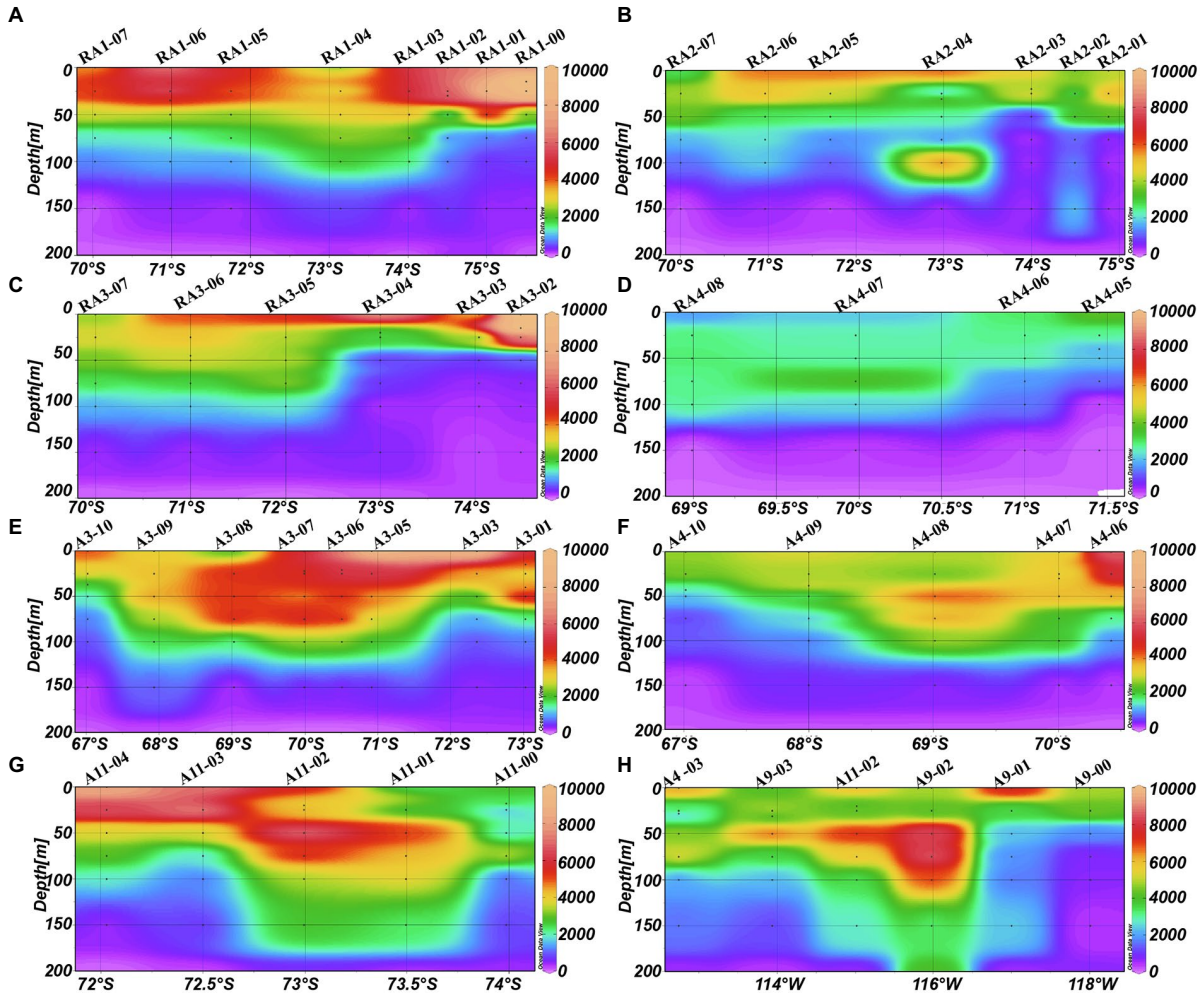
Distributions of picoeukaryotes (cells ml^−1^) in the Amundsen Sea of West Antarctica. **(A)** RA1 transect, **(B)** RA2 transect, **(C)** RA3 transect, **(D)** RA4 transect, **(E)** A3 transect, **(F)** A4 transect, **(G)** A11 transect, and **(H)** A9 transect.

The abundance of picoeukaryotes ranged from 0.01 to 12.95 × 10^3^ cells ml^−1^ in the epipelagic zone (Table 1). In the four transects of the Ross and west Amundsen Seas (RA transects, including RA1, RA2, RA3 and RA4), high abundances of picoeukaryotes were observed in the top 75 m (Figures 4A–E). Whereas in the four transects of the east Amundsen Sea (A transects, including A3, A4, A9 and A11), some high abundances of picoeukaryotes were also found below 100 m, especially in the ASP (A9 and A11 transects; Figures 4A–H).

**Figure 5:**
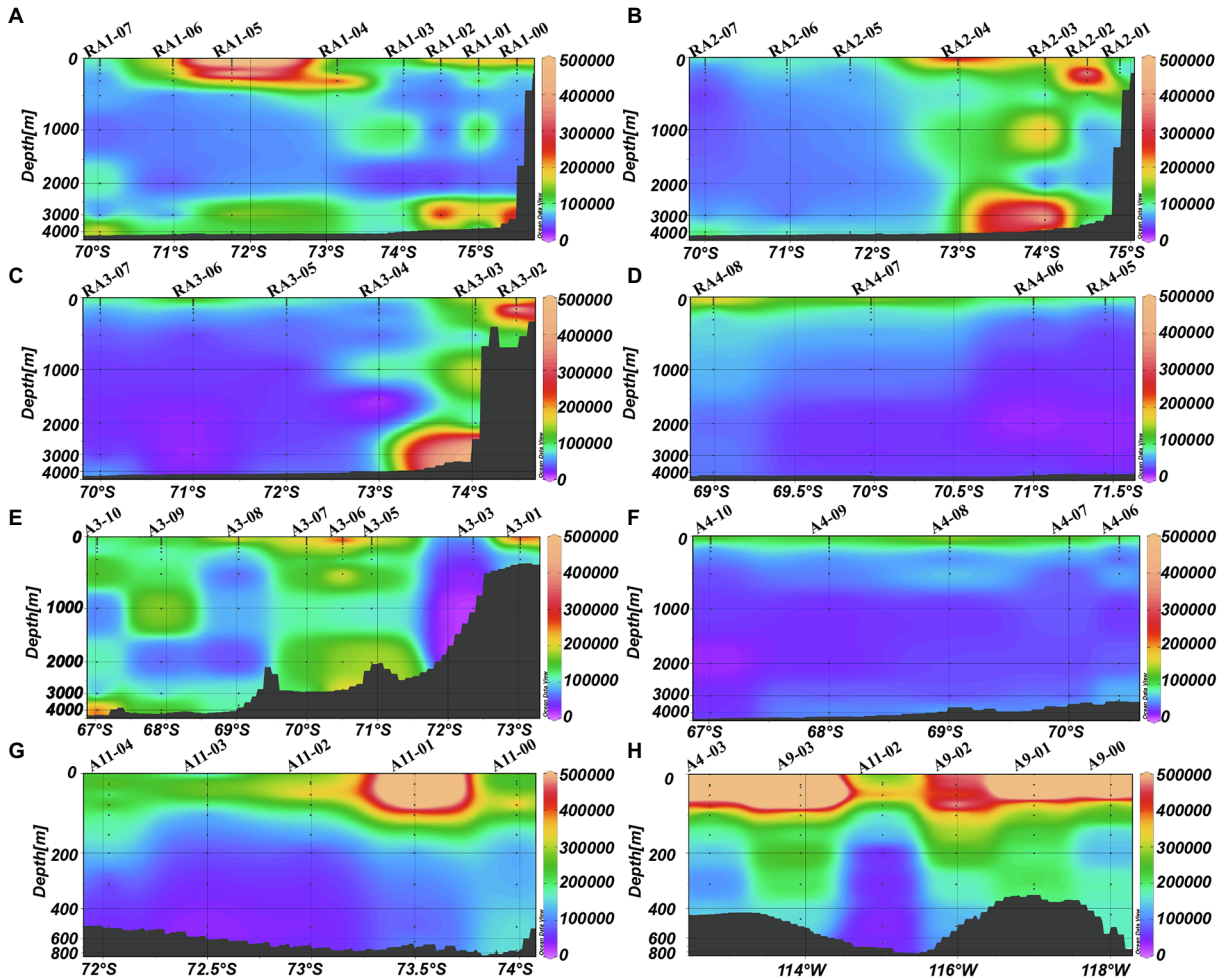
Distributions of heterotrophic prokaryotes (cells ml^−1^) in the Amundsen Sea of West Antarctica. **(A)** RA1 transect, **(B)** RA2 transect, **(C)** RA3 transect, **(D)** RA4 transect, **(E)** A3 transect, **(F)** A4 transect, **(G)** A11 transect, and **(H)** A9 transect.

The abundance of heterotrophic prokaryotes decreased significantly with depth, from 1.82 ± 2.02 × 10^5^ cells ml^−1^ in the epipelagic zone to 0.81 ± 0.88 × 10^5^ cells ml^−1^ in the bathypelagic zone (ANOVA, *p* < 0.001, Table 1). In the meso- and bathypelagic zones, high abundances of heterotrophic prokaryotes were found in the RA1, RA2, RA3, A3, A9 and A11 transects (Figures 5A–C,E,G,H).

### Cluster analysis of virio- and picoplankton distributions in the epipelagic zone

**Figure 6:**
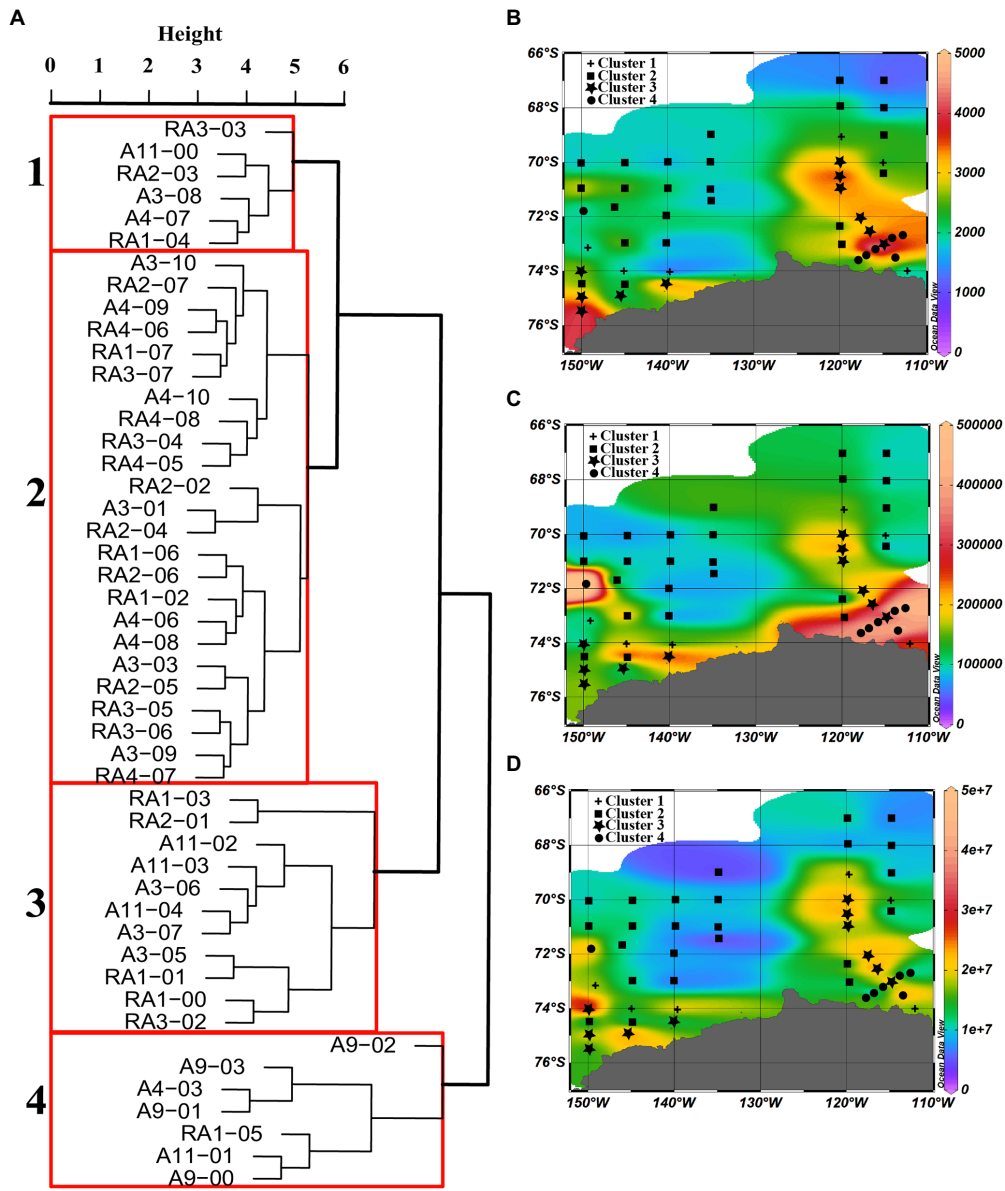
Dendrogram of cluster analysis output of all stations in the epipelagic zone. **(A)** Dissimilarity between the four clusters, and the distributions of water column average abundance of **(B)** picoeukaryotes (cells ml^−1^), **(C)** heterotrophic prokaryotes, and **(D)** viral abundance in the epipelagic zone of stations in each cluster.

Based on a cluster analysis of the microbial abundance (virioplankton, heterotrophic prokaryotes and picoeukaryotes), four clusters were identified (Figure 6A). Significant differences in picoeukaryote, heterotrophic prokaryote and virioplankton abundances between the clusters were observed (*p* < 0.001, Table 2), whereas no significant differences were observed in temperature, salinity, VPR and VEukR. Clusters three and four mainly contained the stations close to the continental shelf, in the transect A3 close to the ASP and in the A9 and A11 transects in the ASP, while cluster two mainly contained oceanic site stations. Cluster one mainly contained stations between the sites in clusters three, four and two (Figure 6). Cluster two was characterized by the lowest abundances of virioplankton, heterotrophic prokaryotes and picoeukaryotes. The abundances of picoeukaryotes and heterotrophic prokaryotes in clusters three and four were higher than those in cluster one, whereas the viral abundances in clusters one and three were higher than that in cluster four (Table 2).

**Table 2:** Environmental factors, viral abundance and ratios between virus and specific picoplankton potential host lineages of each cluster in the epipelagic zone (0–200 m) of the Amundsen Sea of West Antarctica.

	Parameters	Cluster1 (*n* = 45)	Cluster2 (*n* = 180)	Cluster3 (*n* = 82)	Cluster4 (*n* = 51)
Temperature (°C) (*p* = 0.320)	Average	−0.93	−0.88	−1.09	−0.80
	s.d.	1.00	0.96	0.92	1.21
	Median	−1.41	−1.29	−1.52	−1.47
	Min	−1.81	−1.80	−1.81	−1.81
	Max	1.72	1.67	1.57	2.04
Salinity (‰) (*p* = 0.924)	Average	34.01	33.98	33.98	34.00
	s.d.	0.32	0.36	0.34	0.21
	Median	34.09	34.06	34.08	34.05
	Min	33.02	32.23	32.68	33.07
	Max	34.62	34.60	34.65	34.49
DO (mg L^−1^) (*p* = 0.918)	Average	10.30	10.30	10.20	10.13
	s.d.	1.97	1.68	1.74	1.60
	Median	9.87	10.54	9.78	9.25
	Min	5.97	6.12	5.96	7.37
	Max	15.60	13.59	14.05	14.26
Picoeukaryotes (×10^3^ cells ml^−1^; *p* < 0.0001)	Average	1.88	2.07	3.37	3.14
	s.d.	1.54	1.82	3.21	2.19
	Median	2.01	1.76	2.85	2.53
	Min	0.05	0.02	0.01	0.05
	Max	4.72	9.48	12.95	8.48
Heterotrophic prokaryotes (×10^5^ cells ml^−1^; *p* < 0.0001)	Average	1.47	1.11	1.77	4.71
	s.d.	0.82	0.99	1.58	3.23
	Median	1.13	0.90	1.54	4.62
	Min	0.53	0.34	0.14	0.44
	Max	4.23	9.43	13.34	18.22
Viruses (×10^7^ VLPs ml^−1^; *p* < 0.0001)	Average	1.72	0.94	2.27	1.47
	s.d.	0.92	0.57	1.86	1.48
	Median	1.41	0.82	1.66	1.16
	Min	0.30	0.04	0.26	0.00
	Max	4.76	3.61	10.16	6.30
VPR (*p* = 0.237)	Average	151.40	110.84	182.18	69.57
	s.d.	108.58	78.95	193.67	155.54
	Median	124.95	98.24	112.45	21.37
	Min	13.74	7.04	9.50	0.01
	Max	549.08	390.19	1066.70	790.59
VEukR (×10^3^; *p* = 0.081)	Average	57.04	24.35	210.30	15.37
	s.d.	150.55	63.34	1163.64	43.33
	Median	10.68	5.07	9.58	3.72
	Min	0.69	0.62	0.91	0.00
	Max	952.22	633.10	10160.80	286.37

### Relationships among virio-, picoplankton and environmental factors in the epi-, meso- and bathypelagic zones

**Figure 7:**
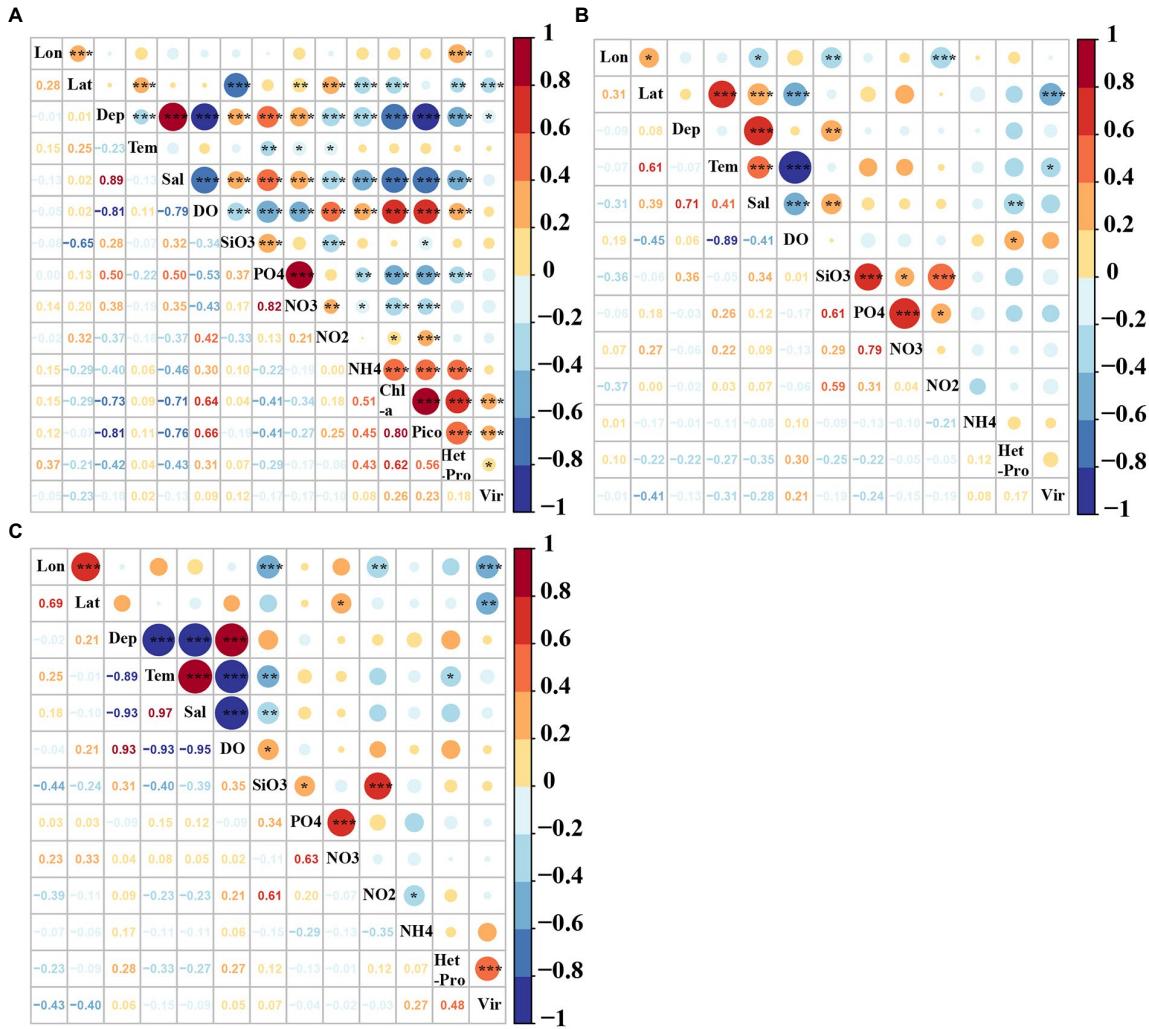
Pearson correlation coefficients among virio-, picoplankton and environmental factors in the **(A)** epi-, **(B)** meso-, and **(C)** bathypelagic zones of the Amundsen Sea of West Antarctica. Lon: Longitude, Lat: Latitude, Tem: Temperature, Sal: Salinity, Pico: Picoeukaryotes, HetPro: Heterotrophic prokaryotes,Vir: Viruses. *Correlation is significant at the 0.05 level (2-tailed), **Correlation is significant at the 0.01 level (2-tailed), ***Correlation is significant at the 0.001 level (2-tailed).

In the epipelagic zone, the abundances of picoeukaryotes, heterotrophic prokaryotes and virioplankton were positively correlated with the Chl-*a*, DO, NO_2_, NH_4_ and longitude, and negatively correlated with latitude, depth, temperature, salinity, SiO_3_, PO_4_ and NO_3_ (*p* < 0.05, Figure 7A). The abundances of viruses, heterotrophic prokaryotes and picoeukaryotes were positively correlated with each other (*p* < 0.05, Figure 7A). In the mesopelagic zone, viral abundance was negatively correlated with temperature and latitude. Heterotrophic prokaryotic abundance was positively correlated with DO, whereas it was negatively correlated with salinity (*p* < 0.05, Figure 7B). In the bathypelagic zone, viral abundance was only positively correlated with heterotrophic prokaryotes whereas it was negatively correlated with longitude and latitude. Heterotrophic prokaryotic abundance was positively correlated with longitude (*p* < 0.05, Figure 7C).

The best variables to explain the variations of virio- and picoplankton abundances in the epi-, meso- and bathypelagic zones were determined by multivariate regression analysis (Table 3). In the epipelagic zone, the variability in viral abundance was mainly explained by Chl-*a* concentration and latitude (*r*^2^ = 0.09). The main predictor variables of the variations in heterotrophic prokaryotic abundance were Chl-*a*, longitude, picoeukaryotes, NO_2_, NH_4_ and PO_4_, which together contributed to 47% of the total variation. Salinity, Chl-*a*, temperature, latitude, heterotrophic prokaryotes, depth, NH_4_, NO_2_, PO_4_ and longitude were the main predictor variables for picoeukaryotic abundance (*r*^2^ = 0.73). In the mesopelagic zone, viral abundance was mostly explained by latitude and SiO_3_ (*r*^2^ = 0.18), and the heterotrophic prokaryotes were only best explained by salinity (*r*^2^ = 0.15). In the bathypelagic zone, heterotrophic prokaryotic abundance, latitude and NH_4_ were the main predictor variables for variations in viral abundance (*r*^2^ = 0.33), and variation in heterotrophic prokaryote abundance was mainly explained by viral abundance and depth (*r*^2^ = 0.31).

**Table 3:** Results of the multivariate regression analysis with forward selection (DISTLM forward) to explain the variability in virio- and picoplankton abundance in the epi- (0–200 m), meso- and bathypelagic zone of the Amundsen of West Antarctica.

Zone	Response variable	Selected variables	Pseudo-*F*	*P*	*r* ^2^	Cumulative
Epipelagic (*n* = 341)	Virioplankton	Chl-*a*	25.55	0.001	0.07	0.07
		Latitude	7.39	0.003	0.02	0.09
	HeteProk	Chl-*a*	137.08	0.001	0.29	0.29
		Longitude	49.84	0.001	0.09	0.38
		Picoeukaryotes	22.27	0.001	0.04	0.42
		NO_2_	18.70	0.001	0.03	0.45
		NH_4_	10.83	0.001	0.02	0.47
		PO_4_	4.00	0.044	0.01	0.47
	Picoeukaryotes	Salinity	219.85	0.001	0.39	0.39
		Chl-*a*	75.27	0.001	0.11	0.50
		Temperature	105.56	0.001	0.12	0.62
		Latitude	47.17	0.001	0.05	0.67
		HeteProk	24.51	0.001	0.02	0.69
		Depth	24.50	0.001	0.02	0.71
		NH_4_	4.65	0.02	0.00	0.72
		NO_2_	4.85	0.02	0.00	0.72
		PO_4_	7.25	0.007	0.01	0.73
		Longitude	4.11	0.029	0.00	0.73
Mesopelagic (*n* = 132)	Virioplankton	Latitude	22.40	0.001	0.15	0.15
		SiO_3_	5.14	0.025	0.03	0.18
	HeteProk	Salinity	23.13	0.001	0.15	0.15
Bathypelagic (*n* = 102)	Virioplankton	HeteProk	27.27	0.001	0.21	0.21
		Latitude	11.61	0.002	0.08	0.30
		NH_4_	4.93	0.039	0.03	0.33
	HeteProk	Virioplankton	27.07	0.001	0.21	0.21
		Depth	13.51	0.001	0.09	0.31

HetProk, Heterotrophic prokaryotes; Pseudo-*F* and the *p* values were obtained by permutation (*n* = 9,999).

### Relationships between virio-, picoplankton and environmental factors in the four clusters

**Figure 8:**
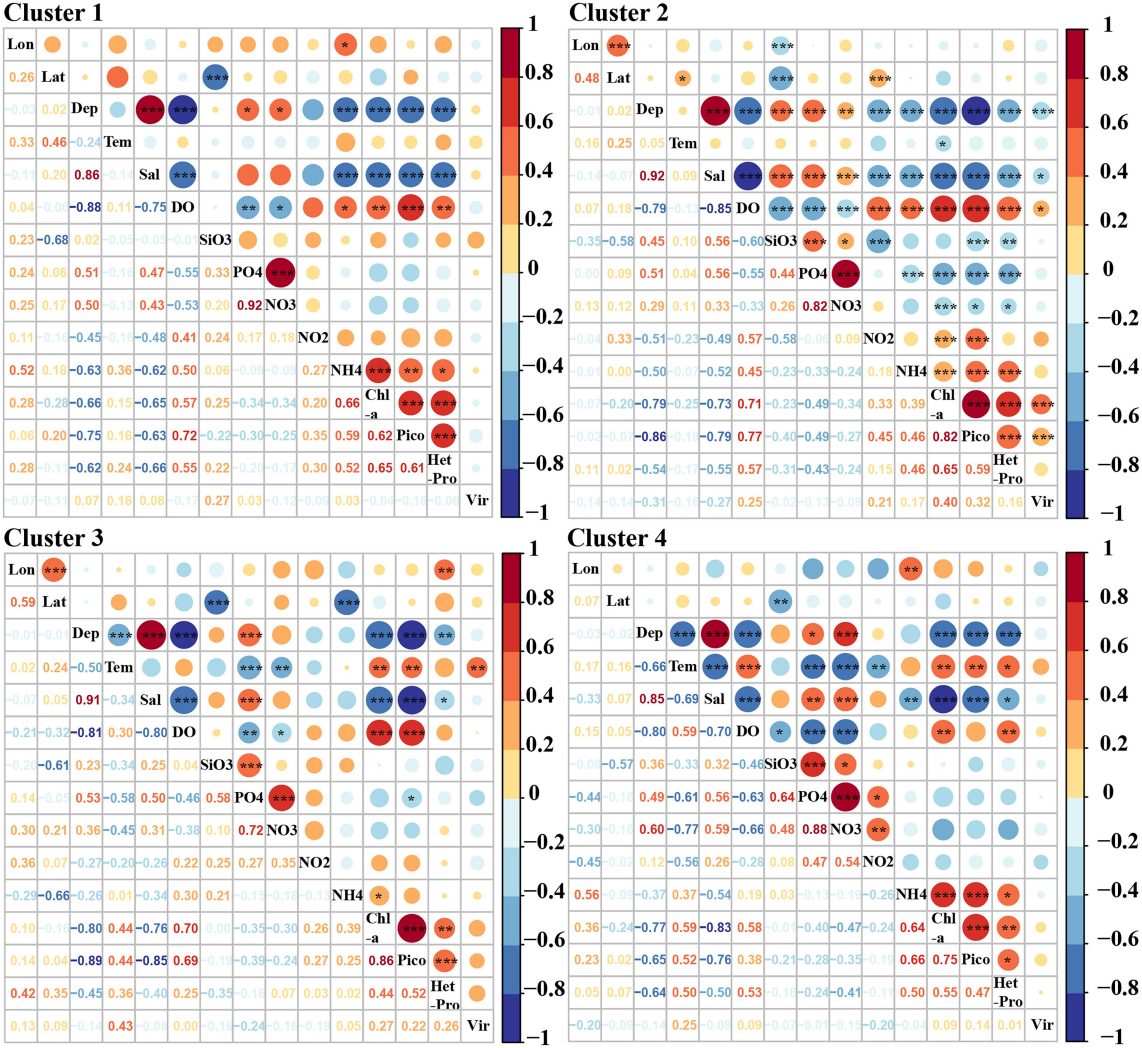
Pearson correlations among virio-, picoplankton and environmental parameters in each cluster. Lon: Longitude, Lat: Latitude, Tem: Temperature, Sal: Salinity, Pico: Picoeukaryotes, HetPro: Heterotrophic prokaryotes,Vir: Viruses. * Correlation is significant at the 0.05 level (2-tailed), ** Correlation is significant at the 0.01 level (2-tailed), *** Correlation is significant at the 0.001 level (2-tailed).

Significant correlations between picoplankton and environmental factors were observed in all four clusters. Picoeukaryotes and heterotrophic prokaryotic abundances in particular, were positively correlated with Chl-*a* (Figure 8, *p* < 0.01). A significant positive relationship was also found between picoeukaryotic and heterotrophic prokaryotic abundance in all four clusters (Figure 8, *p* < 0.01). However, viral abundance was only positively correlated with picoeukaryotic abundance and Chl-*a* in cluster two (Figure 8, *p* < 0.001).

Multivariate regression analysis was used to predict the best variables to explain the variations in the abundance of virio- and picoplankton in each cluster. The variability in viral abundance was only explained by longitude (*r*^2^ = 0.08) in cluster one, by picoeukaryotic abundance (*r*^2^ = 0.15) in cluster two, by temperature and picoeukaryotic abundance (*r*^2^ = 0.19) in cluster three, and by longitude, latitude and NH_4_ (*r*^2^ = 0.35) in cluster four (Table 4). The variability of heterotrophic prokaryotic abundance was explained by Chl-*a* and picoeukaryotes in cluster one (*r*^2^ = 0.60), by Chl-*a*, picoeukaryotes, NH_4_, depth, latitude, NO_2_, NO_3_ and PO_4_ (*r*^2^ = 0.56) in cluster two, by picoeukaryotes, longitude and NO_2_ (*r*^2^ = 0.37) in cluster three, and only by Chl-*a* (*r*^2^ = 0.32) in cluster four, respectively. Autotrophic picoeukaryotic abundance was mainly explained by DO, NH_4_, SiO_3_, NO_3_, salinity, heterotrophic prokaryotes, virioplankton in in cluster one (*r*^2^ = 0.88), by salinity, temperature, Chl-*a*, NO_2_, heterotrophic prokaryotes, depth, longitude and SiO_3_ (*r*^2^ = 0.80) in cluster two, by DO, latitude, temperature, Chl-*a* and NH_4_ in cluster three (*r*^2^ = 0.80), and by Chl-*a*, temperature, salinity, NH_4_, SiO_3_ and NO_3_ in cluster four (*r*^2^ = 0.76), respectively (Table 4).

**Table 4:** Results of the multivariate regression analysis with forward selection to explain the variability in virio- and picoplankton abundance of each cluster in the epipelagic zone.

Cluster	Response variable	Selected variables	Pseudo-*F*	*P*	*r* ^2^	Cumulative
1	Virioplankton	Longitude	4.83	0.034	0.08	0.08
	HeteProk	Chl-*a*	40.66	0.001	0.49	0.49
		Picoeukaryotes	12.09	0.005	0.11	0.60
	Picoeukaryotes	DO	44.11	0.001	0.51	0.51
		NH_4_	12.60	0.001	0.11	0.62
		SiO_3_	25.49	0.001	0.15	0.77
		NO_3_	16.89	0.001	0.07	0.84
		Salinity	5.86	0.015	0.02	0.86
		HeteProk	4.24	0.036	0.01	0.87
		Virioplankton	3.96	0.049	0.01	0.88
2	Virioplankton	Picoeukaryotes	30.12	0.001	0.15	0.15
	HeteProk	Chl-*a*	98.60	0.001	0.37	0.37
		Picoeukaryotes	21.27	0.001	0.07	0.44
		NH_4_	8.39	0.005	0.03	0.47
		Depth	8.39	0.01	0.03	0.49
		Latitude	6.54	0.021	0.02	0.51
		NO_2_	4.74	0.028	0.01	0.53
		NO_3_	5.00	0.026	0.01	0.54
		PO_4_	5.55	0.028	0.02	0.56
	Picoeukaryotes	Salinity	144.65	0.001	0.46	0.46
		Temperature	86.02	0.001	0.18	0.65
		Chl-*a*	25.44	0.001	0.05	0.69
		NO_2_	33.23	0.001	0.05	0.74
		HeteProk	11.82	0.001	0.02	0.76
		Depth	13.58	0.001	0.02	0.78
		Longitude	9.50	0.003	0.01	0.79
		SiO_3_	5.70	0.014	0.01	0.80
3	Virioplankton	Temperature	12.56	0.002	0.14	0.14
		Picoeukaryotes	5.15	0.026	0.05	0.19
	HeteProk	Picoeukaryotes	20.26	0.001	0.20	0.20
		Longitude	14.45	0.001	0.12	0.33
		NO_2_	5.60	0.017	0.05	0.37
	Picoeukaryotes	DO	83.24	0.001	0.51	0.51
		Latitude	27.61	0.001	0.13	0.64
		Temperature	20.54	0.001	0.08	0.71
		Chl-*a*	23.60	0.001	0.07	0.78
		NH_4_	6.70	0.007	0.02	0.80
4	Virioplankton	Longitude	3.53	0.054	0.08	0.08
		Latitude	10.35	0.003	0.19	0.26
		NH_4_	5.64	0.028	0.09	0.35
	HeteProk	Chl-*a*	19.89	0.001	0.32	0.32
	Picoeukaryotes	Chl-*a*	36.90	0.001	0.47	0.47
		Temperature	8.75	0.003	0.09	0.56
		Salinity	9.38	0.008	0.08	0.64
		NH_4_	6.42	0.017	0.05	0.69
		SiO_3_	5.06	0.027	0.04	0.73
		NO_3_	4.31	0.039	0.03	0.76

HetProk, Heterotrophic prokaryotes; Pseudo-*F* and the *P* values were obtained by permutation (*n* = 9,999).

## Discussion

The Amundsen Sea is a climate-sensitive region that has experienced the most rapid decline in sea ice cover over the past several decades in the Antarctica (Walker et al., 2007). The ASP is one of the largest and most productive coastal polynyas in the Southern Ocean (Arrigo and van Dijken, 2003). There have been few studies focusing on the abundance and distribution of virio- or picoplankton in Antarctic polynyas (Hyun et al., 2016; Williams et al., 2016). These studies were only sampled above 100 m of the water column. To address this shortcoming, we report here on the distribution and abundance of virio- and picoplankton and their relationships with key environmental factors in the Amundsen Sea, including the ASP.

### Distributions of epipelagic pico- and virioplankton and their relationships with environmental factors

The abundances of picoeukaryotes, heterotrophic prokaryotes and virioplankton in the epipelagic zone in summer are similar to those documented in previous reports from Antarctica (Table 1; Church et al., 2003; Pearce et al., 2007; Sawstrom et al., 2007; Thomson et al., 2010; Lin et al., 2012; Hyun et al., 2016; Liang et al., 2016; Williams et al., 2016; Han et al., 2022). In particular, in the same region in ASP between 14 December 2010 and 8 January 2011, the viral and bacterial abundance ranged from 0.1 to 8.2 × 10^9^ VLPs L^−1^ and 0.1 to 4.0 × 10^9^ cells L^−1^, respectively (Williams et al., 2016). A Korean Amundsen Sea expedition, from February 9 to March 10 in 2012, also reported that bacterial abundances in the mixed layer were higher in the polynya (4.77–11.83 × 10^5^ cells ml^−1^) than on the outer shelf (1.44–3.47 × 10^5^ cells ml^−1^), ice shelf (1.64–5.36 × 10^5^ cells ml^−1^) or open sea (0.37–0.60 × 10^5^ cells ml^−1^; Hyun et al., 2016). Based on the abundances of autotrophic picoeukaryotes, heterotrophic prokaryotes and virioplankton in the epipelagic zone, four clusters were identified (Figure 6A). Pearson correlation and multivariate regression analyses showed that picoeukaryotic abundance was closely linked to Chl-*a* in all four clusters, this strongly implies that picoeukaryotic abundance is associated with the presence of ice-edge blooms (Figure 8; Table 4). In the Southern Ocean, autotrophic picoplankton can contribute 20–40% of the chlorophyll *a* biomass (Hewes, 2009; Vanzan et al., 2015) and up to 74% of the phytoplankton biomass in the Drake Passage/Bransfield Strait (Agawin et al., 2002). Stations in clusters one and two, with lower picoeukaryotic and heterotrophic prokaryotic abundances, were mainly located in oceanic waters and the junction between oceanic and offshore waters (Figure 6). Unlike cluster two, which had the lowest viral abundance, cluster one had a moderately high viral abundance. The reason for this high viral abundance is still unclear, but the accompanying data suggests several possible explanations. The stations in cluster one are mainly located near the ice edges or areas of recent sea ice retreat (Supplementary Figures S1, S6). The moderate concentration of Chl-*a* is likely to have been contributed by nano-phytoplankton groups other than picoeukaryotes, e.g., diatoms, at RA2-03 and RA3-03 stations (Hao et al., Unpubl.), and the moderate picoeukaryotic abundances at RA1-04, A3-08 and A4-07 stations (Figures 4A,E,F) in cluster one might suggest these areas were in a post-bloom development stage where the decline in the picoeukaryotic communities was due to grazing and/or senescence (Thomson et al., 2010). Viral abundance increased significantly in the post-bloom stages, probably after phytoplankton cell lysis (Suttle et al., 1990).

The high abundance of picoeukaryotes and high concentrations of Chl-*a* in clusters three and four strongly suggests the presence of ice-edge algal blooms (Figure 6, Hao et al., Unpubl.). Stations in clusters three and four were mainly either near the coast, near upwelling Circumpolar Deep Water (CDW) or in the ASP (Figures 6; Supplementary Figure S5). Iron (Fe) has been shown to be a limiting nutrient for phytoplankton growth in much of the Southern Ocean (Debaar et al., 1995). Although iron was not measured in this study, several reports have determined that an adequate iron supply from diverse sources, including melting from the base of glaciers, upwelling CDW in ASP (Gerringa et al., 2012; Sherrell et al., 2015) and shelf sediments (de Jong et al., 2013; Hatta et al., 2013) probably stimulated and sustained the growth of phytoplankton in these areas. Similarly, the high abundance of heterotrophic prokaryotes and their strong correlation with picoeukaryote abundance and Chl-*a* concentration in these two clusters (Table 2; Figure 8), suggests that a large amount of organic carbon was provided by phytoplankton and this promoted the growth of heterotrophic prokaryotes (Granéli et al., 2004; Pearce et al., 2007; Hyun et al., 2016; Serret et al., 2016). Viral abundances were also high in cluster three, whereas they were relatively low in cluster four; viruses were not significantly correlated with heterotrophic prokaryotes, picoeukaryotes or Chl-*a.* These results are consistent with previous studies (Thomson et al., 2010) and indicate that viruses may adopt different life styles during blooms compared to non-bloom situations. The high VPR and VEukR in cluster three suggest that the infection rates were commonly high but variable and that there may be a time delay between viral lysis and the peak abundance of their hosts (Arkhipova et al., 2018). Unlike cluster three, the lower viral abundance and the lowest VPR and VEukR in cluster four may support the viral “piggyback the winner” life style, which proposes that viruses exploit their hosts through lysogeny rather than killing their hosts when they achieve high densities (Knowles et al., 2016).

### Vertical distributions of virio- and picoplankton and their relationships with environmental factors

As expected, the vertical distribution patterns of virio- and picoplankton abundance in the Amundsen Sea were similar to those elsewhere in Antarctic ocean areas (Yang et al., 2014; Liang et al., 2016; Han et al., 2022). In the epipelagic zone, viral abundance was significantly correlated with autotrophic picoeukaryotes and Chl-*a* (Figure 7), as in previous studies (Danovaro et al., 2011; Han et al., 2022). In the meso- and bathypelagic zones, the upwelling of CDW (Supplementary Figure S5; Thomson et al., 2010) and sinking particulate organic carbon (POC; Taylor et al., 2003; Bochdansky et al., 2010; Yang et al., 2014) have been proposed as the main factors influencing heterotrophic prokaryotic and viral distributions. As in Prydz Bay (Liang et al., 2016) and the Cooperation Sea, Antarctica (Han et al., 2022), high abundances of heterotrophic prokaryotes and viruses were found in the meso- and bathypelagic zones of ice-edge bloom areas (Figures 3, 5). Furthermore, the dynamics of potential viral host cells, especially heterotrophic prokaryotes (Magagnini et al., 2007; De Corte et al., 2010; Liang et al., 2016), also influences the vertical distribution of viruses. The results of the Pearson and the multivariate regression analysis both strongly suggest that there is a close link between virus and heterotrophic prokaryote abundance in the bathypelagic zone of the Amundsen Sea (Figure 7; Table 3).

The high abundance of viruses and elevated VPR found in the meso- and bathypelagic zones (Figure 3; Table 1) is likely due to the strong vertical transport of viruses attached to sinking particles with subsequent dissociation at depth (Taylor et al., 2003; Bochdansky et al., 2010). About 14–60% primary production has been reported to be exported from the euphotic zone to the mesopelagic zone in the ASP (Yager et al., 2012; Kim et al., 2014). However, evidence of minimal carbon sequestration in the productive ASP was also investigated in a companion study (Lee et al., 2017). This study found that most of the POC exported from the surface mixed layer was converted to non-sinking forms such as fine suspended POC and/or as dissolved carbon, either dissolved inorganic carbon (DIC) or dissolved organic carbon (DOC) before reaching the bottom (Lee et al., 2017). High bacterial respiration may also be an important factor influencing POC flux (Ducklow et al., 2015) In this study, a high abundance of heterotrophic prokaryotes was also found in the mesopelagic zone (Figure 3H), suggesting the presence of bacterial respiration. This high heterotrophic prokaryotic abundance may have induced the high viral abundance (Figures 5G,H). Another factor potentially responsible for the minimal carbon sequestration in the ASP is the intrusion of CDW deep onto the Amundsen shelf along the bottom, with the carbon-laden water flowing off the shelf into the upper layers (Petty et al., 2013; Lee et al., 2017). Thus, the water masses and currents, especially the CDW may also influence the distribution of virioplankton in the deep sea. Viruses can attach to the POC from the bottom and be transported by the CDW to the upper layers and then dissociated. An oceanographic cruise carried out in the Ross Sea during the summer 2005/06 found that different water masses, including Antarctic Surface Waters (AASW), High Salinity Shelf Water (HSSW), Ice Shelf Water (ISW), Antarctic Bottom Water (AABW) and Circumpolar Deep Water (CDW), all contained diverse bacterial communities (Celussi et al., 2010). Although there have no previous reports of viruses in Antarctic water masses, a study in the Nordic Seas found that the distribution patterns of Caudovirales and major giant nucleocytoplasmic large DNA viruses (NCLDVs) were a reflection of the community structure of their hosts in the corresponding water masses and currents (Gao et al., 2021). We propose here that the high viral abundance in meso- and bathypelagic zones are influenced by the upwelling CDW. In addition, zooplankton fecal pellets are an important component of the POC export to depth (Turner, 2015). Gleiber et al. found that fecal pellet POC was the dominant component of total organic carbon flux with significantly higher pellet flux (67%) in summer (November to April) than in winter (May to October; 34%) in the western Antarctic Peninsula (Gleiber et al., 2012) and there was significant evidence of incorporation of picoplankton into sinking fecal pellets (Lomas and Moran, 2011; Amacher et al., 2013). The high abundances of picoeukaryotes were found below 100 m in the ASP, which may also suggest strong vertical transport of POC (Figure 4). Although picoeukaryotes were not detected below 200 m, the high picoeukaryotic abundance in the upper layer may have sustained zooplankton grazing with, a resultant fecal pellet flux, which may have included attached viruses and bacteria, which were then exported to the deep sea as part of the POC. In summary, although POC data were not available to supported this suggestion, high abundances of pico- and heterotrophic prokaryotes and high concentrations of Chl-*a* in the epipelagic zone at these stations, combined the presence of upwelling CDW, was associated with a high abundance of viruses (Figures 3–5; Hao et al., Unpubl.). The high biomass in the upper layers is likely to have contributed to a high vertical export of POC, having a crucial role in the vertical transport of viruses (Smetacek, 2000; Bochdansky et al., 2010; De Corte et al., 2010, 2012; Herndl and Reinthaler, 2013; Liang et al., 2016, 2017; Han et al., 2022). The virus family *Phycodnaviridae* was abundant in deep waters (>3,000 m) of Prydz Bay (Gong et al., 2018). This virus family is associated with marine microalgae, suggesting that heterotrophic prokaryotes and viruses induced by surface algal blooms can be transported to depth (Jiao et al., 2010, 2018).

## Conclusion

High abundances of picoeukaryotes, heterotrophic prokaryotes and virioplankton were found in the epipelagic zone of areas associated with ice-edge blooms areas near the coast and in the ASP. Picoeukaryotes and heterotrophic prokaryotes abundances were closely linked to the Chl-*a* concentration. There may be a time-lag between viruses and their potential hosts. High abundances of heterotrophic prokaryotes and viruses in the meso- and bathypelagic zones are likely to be dependent on the high primary production at the ice-edge, induced by nutrient input from the CDW upwelling and iron supply from the melting sea ice, and on the subsequent sinking of POC and release of viruses.

This study provides an initial insight into the distribution and role of the viruses during edge algal bloom in and around the ASP of Antarctica. In the future, community structure and function analysis is required to better understand the viral dynamics and virus–host interactions, as well as the ecological roles of virio- and picoplankton in the biogeochemical cycles during the edge blooms in Antarctica.

## Data availability statement

The original contributions presented in the study are included in the article/Supplementary material, further inquiries can be directed to the corresponding authors.

## Author contributions

JH, YL, and MW contributed to conception and design of the study. MH and GLu collected the samples. MH, GLu, HS, XC, GLi, YS, FG, and HY performed the experiments. JZ provided the nutrient data. QH provided the chlorophyll data. MH, YL, MW, and AM analyzed the data. YS, WM, and LW gave suggestions to the manuscript. MH wrote the first draft of the manuscript. JH, YL, AM, and MW contributed to the manuscript revision. All authors contributed to the article and approved the submitted version.

## Funding

This work was supported by the Ministry of Natural Resources of the People’s Republic of China, the program of the Impact and Response of Antarctic Seas to Climate Change (IRASCC 01-02, 02-01), the National Key Research and Development Program of China (no. 2018YFC1406704), the Fundamental Research Funds for the Central Universities (nos. 202072002, 202072001, and 201812002), National Natural Science Foundation of China (nos. 42120104006, 41976117, 42176111, and 42188102).

## Conflict of interest

The authors declare that the research was conducted in the absence of any commercial or financial relationships that could be construed as a potential conflict of interest.

## Publisher’s note

All claims expressed in this article are solely those of the authors and do not necessarily represent those of their affiliated organizations, or those of the publisher, the editors and the reviewers. Any product that may be evaluated in this article, or claim that may be made by its manufacturer, is not guaranteed or endorsed by the publisher.

## Footnotes

^1^ https://seaice.uni-bremen.de/

^2^ https://www.oceancolour.org/

^3^ http://www.R-project.org/
