# Supplementary material for: Distributions and relationships of virio- and picoplankton in the epi-, meso- and bathypelagic zones of the Amundsen Sea, West Antarctica during the austral summer
Source: Front Microbiol. 2022 Jul 27;13:941323. doi: 10.3389/fmicb.2022.941323 (PMC9363919; doi:10.3389/fmicb.2022.941323)
Supplement: Supplementary file 1 [file Image_1.PDF]

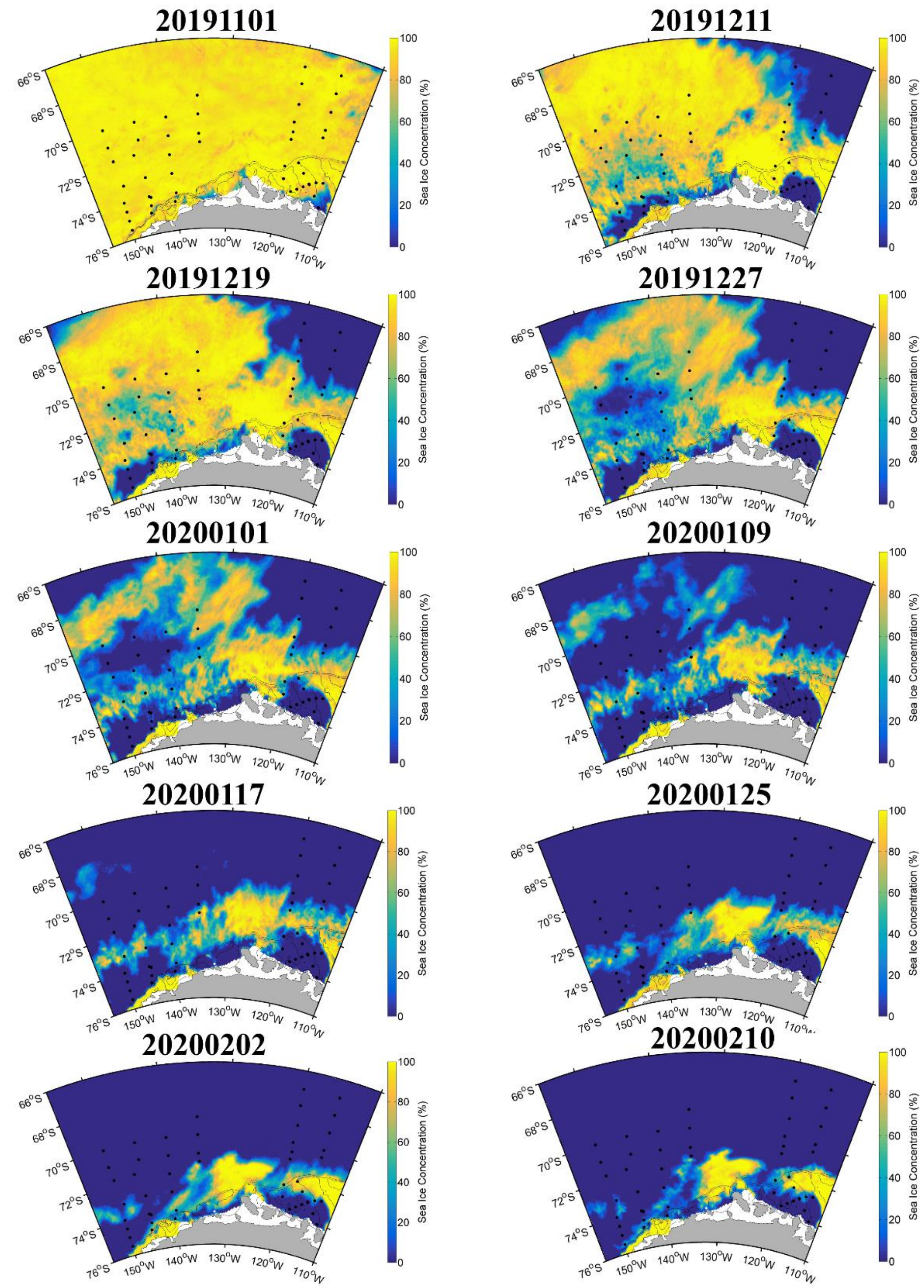

FIGURE S1. Eight days averaged sea ice concentration before and during the observation period in the Amundsen Sea of West Antarctica. The data were obtained from the AMSR2 dataset at the University of Bremen, with a spatial resolution of 6.25 km× 6.25 km.

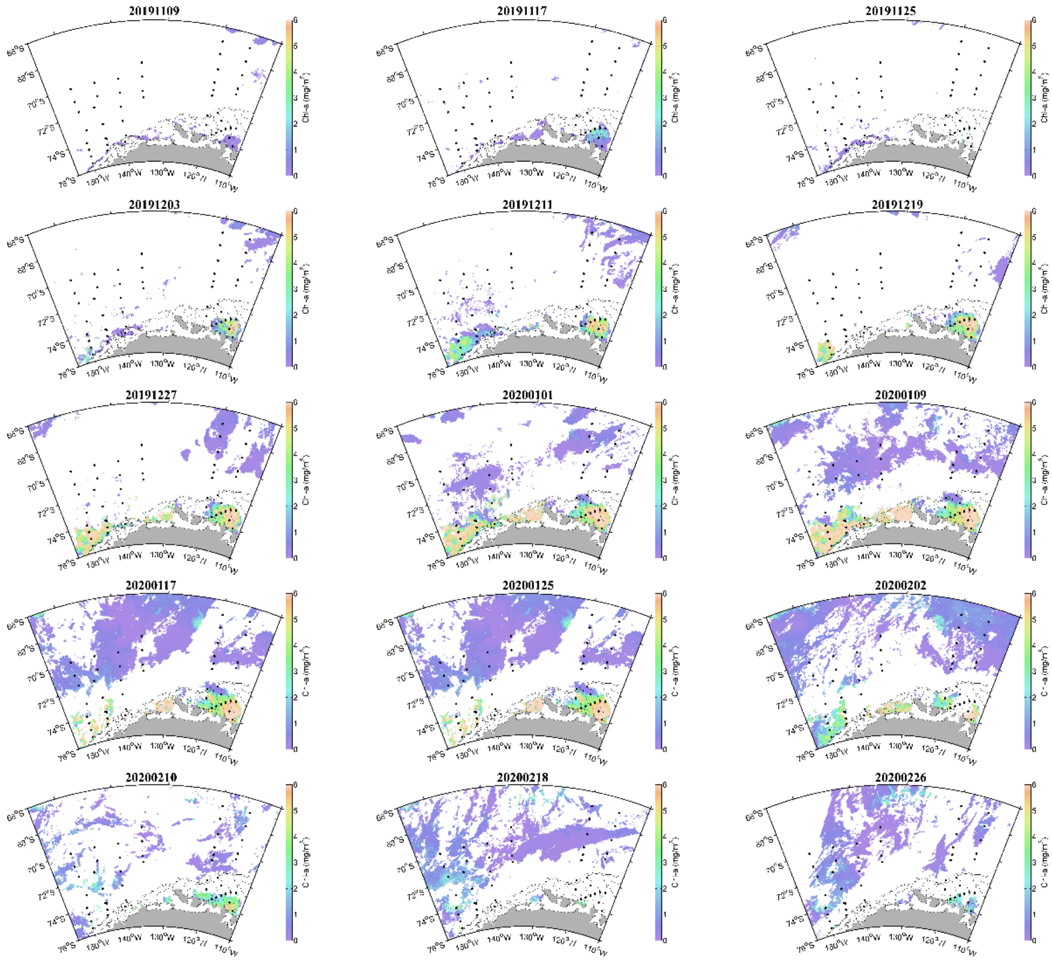

FIGURE S2. Eight days averaged Chl-*a* concentration before and during the observation period in the Amundsen Sea of West Antarctica. The data were retrieved from the products generated by the Ocean Colour component of the European Space Agency Climate Change Initiative project.

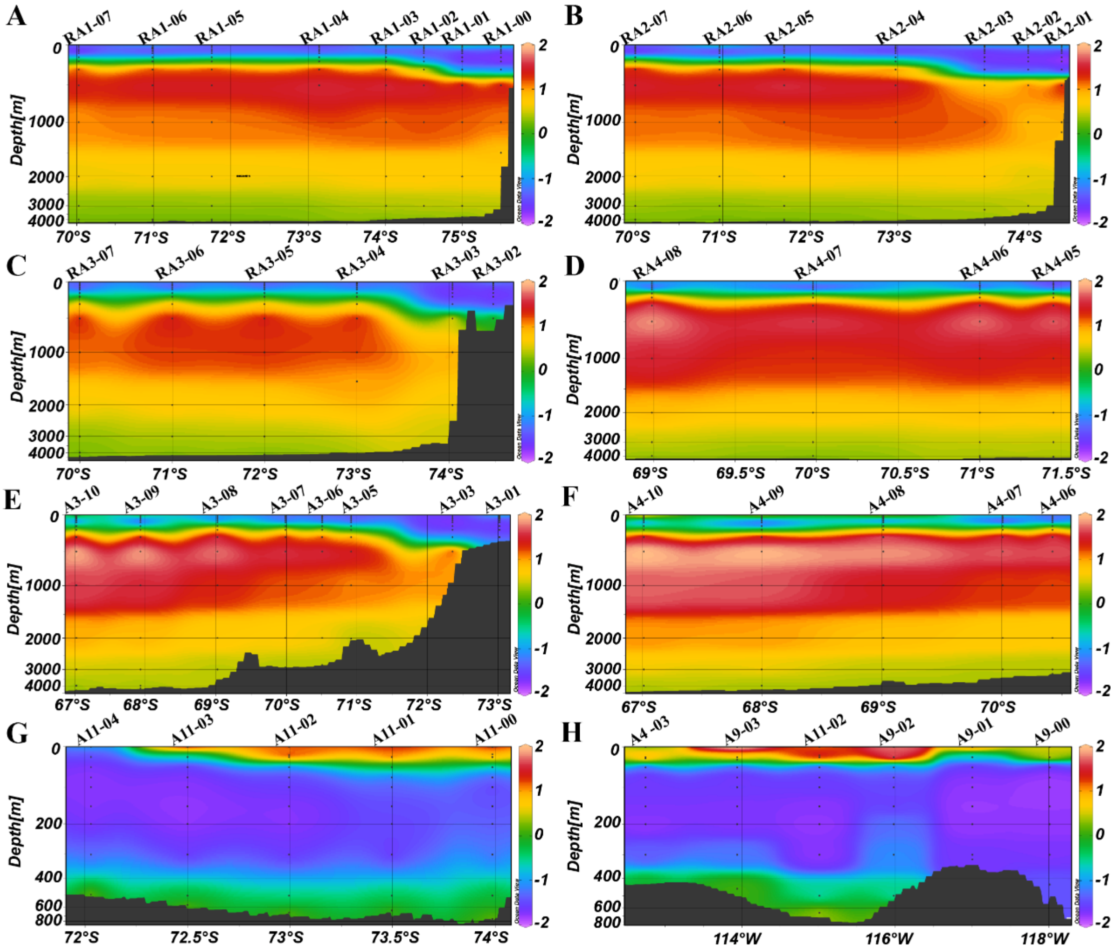

FIGURE S3. Distributions of temperature (°C) in the Amundsen Sea of West Antarctica. **(A)** RA1 transect, **(B)** RA2 transect, **(C)** RA3 transect, **(D)** RA4 transect, **(E)** A3 transect, **(F)** A4 transect, **(G)** A11 transect, and **(H)** A9 transect.

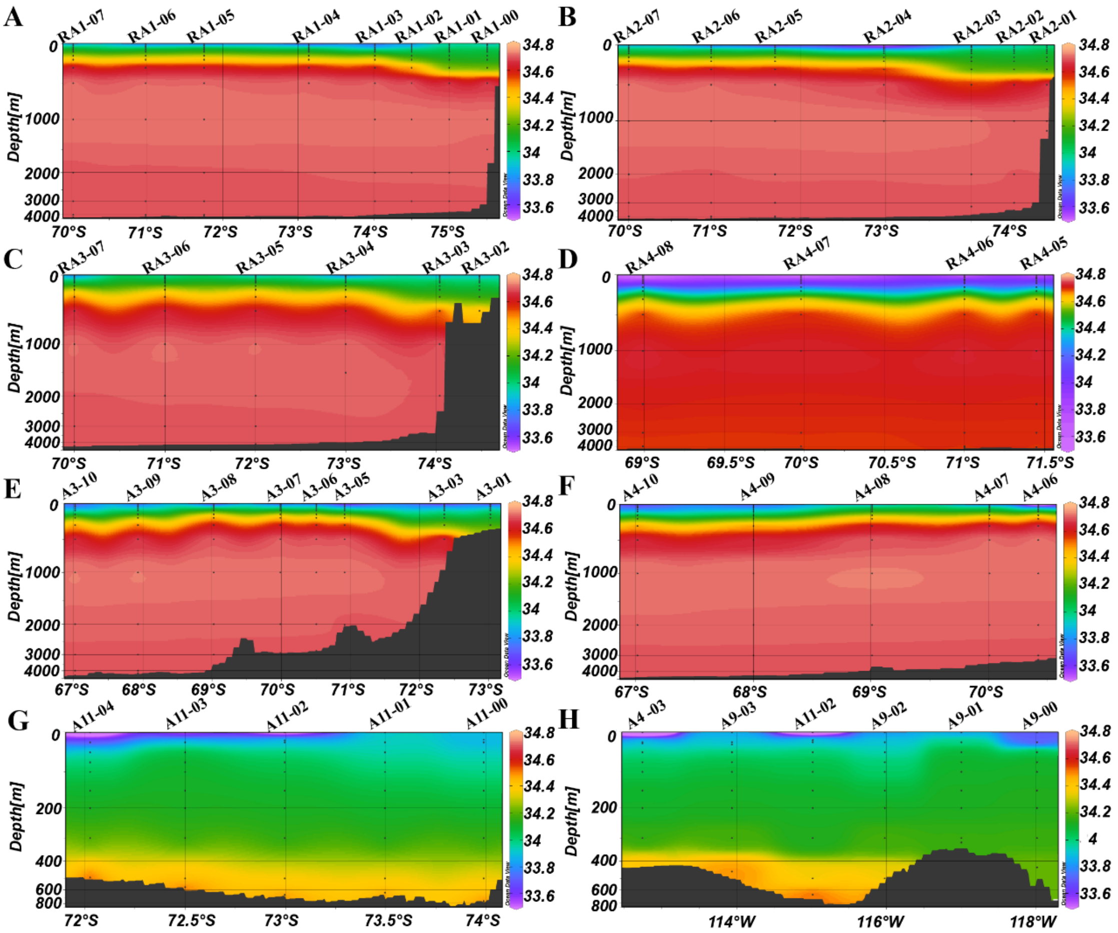

FIGURE S4. Distributions of salinity (‰) in the Amundsen Sea of West Antarctica. **(A)** RA1 transect, **(B)** RA2 transect, **(C)** RA3 transect, **(D)** RA4 transect, **(E)** A3 transect, **(F)** A4 transect, **(G)** A11 transect, and **(H)** A9 transect.

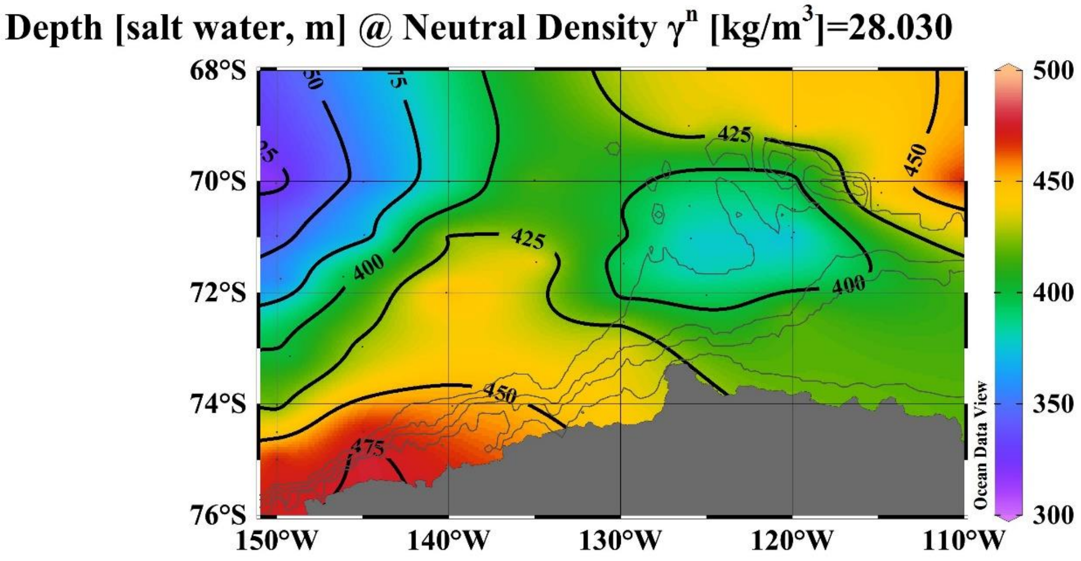

FIGURE S5. The depth of neutral density surface 28.03 kg·m^{-3} in the Amundsen Sea of West Antarctica.
